# Effects of Land Use Change and Seasonality of Precipitation on Soil Nitrogen in a Dry Tropical Forest Area in the Western Llanos of Venezuela

**DOI:** 10.1155/2014/514204

**Published:** 2014-12-31

**Authors:** Ana Francisca González-Pedraza, Nelda Dezzeo

**Affiliations:** ^1^Universidad Nacional Experimental Sur del Lago “Jesús María Semprum” (UNESUR), Programa Ingeniería de la Producción Agropecuaria, Laboratorio de Suelos, Santa Bárbara, Municipio Colón 5148, Estado Zulia, Venezuela; ^2^Instituto Venezolano de Investigaciones Científicas (IVIC), Centro de Ecología, Laboratorio de Ecología de Suelos, Km 11, Carretera Panamericana, Altos de Pipe 1020, Estado Miranda, Venezuela

## Abstract

We evaluated changes of different soil nitrogen forms (total N, available ammonium and nitrate, total N in microbial biomass, and soil N mineralization) after conversion of semideciduous dry tropical forest in 5- and 18-year-old pastures (YP and OP, resp.) in the western Llanos of Venezuela. This evaluation was made at early rainy season, at end rainy season, and during dry season. With few exceptions, no significant differences were detected in the total N in the three study sites. Compared to forest soils, YP showed ammonium losses from 4.2 to 62.9% and nitrate losses from 20.0 to 77.8%, depending on the season of the year. In OP, the ammonium content increased from 50.0 to 69.0% at the end of the rainy season and decreased during the dry season between 25.0 and 55.5%, whereas the nitrate content increased significantly at early rainy season. The net mineralization and the potentially mineralizable N were significantly higher (*P* < 0.05) in OP than in forest and YP, which would indicate a better quality of the substrate in OP for mineralization. The mineralization rate constant was higher in YP than in forest and OP. This could be associated with a reduced capacity of these soils to preserve the available nitrogen.

## 1. Introduction

Soil nitrogen (N) is a key element in primary productivity and soil fertility of ecosystems [1, 2]. N dynamics in tropical dry forest have been related to the rapid rate of mineralization, high available mineral concentrations, and the low efficiency of plant nutrient use [3–5]. Great effort has been dedicated to understand the dynamics of this element after clear-cutting and burning of these forests [1, 6].

The N mineralization involves a series of actions mainly mediated by soil microorganisms [7]. Therefore, this is a sensitive process to disturbance in most forest ecosystems [1, 6]. In a tropical forest, N mineralization depends on the amount and type of organic matter and microbial activity, as well as on soil physicochemical properties and soil moisture content [8].

When the tropical dry forest is cut down and burned to be converted into pasture, the soil temperature increases, causing rapid ammonification and nitrification. Consequently, great losses of N by volatilization, erosion, and leaching occur. Additionally, vegetation absorption and microorganism immobilization are insufficient to prevent these losses [9]. Later, during the early stages of revegetation under pasture, the mineralization and nitrification rates are lower than those in the original forest [5, 10].

The tropical dry forest has been considered one of the world's most threatened ecosystems [11–14]. However, there is little information about land use changes effects for this ecosystem. Most of the studies on this topic have been conducted in rainforest areas [4, 5, 10, 15–20].

Tropical dry forests are characterized by the marked seasonality of rainfall that influences its primary productivity [2, 13, 21]. In Venezuela, this ecosystem represents the most important life zone of land and includes large areas covered by dense seasonal dry forests developed on relatively fertile soils. These forests have been subjected to a high pressure of use associated with population growth and the expansion of the agricultural frontier. After tree cutting, one of the main uses has been the establishing of pastures. However, there is no detailed information about changes occurred in the soil nitrogen once the forests have been cleared and converted into pasture. This information is necessary to anticipate deforestation consequences and to design effective pasture management.

The objective of this research is to evaluate changes in the total N, soil available N (ammonium and nitrate), total N in microbial biomass, and soil N mineralization due to transformation of semideciduous tropical dry forest into pastures in an area located in the western Llanos, Venezuela.

## 2. Materials and Methods

### 2.1. Study Site

The study area was located in the western Llanos of Venezuela, at approximately 120 m asl, between 40°01′10′′ and 40°59′10′′ north latitude and 91°57′30′′ and 91°25′18′′ west longitude. The average annual rainfall in this region is 1243.7 mm, with a rainy season from April to December and a dry season from January to March. The average annual temperature is 26.8°C, with a maximum of 28.9°C between March and April and a minimum of 25.5°C between December and January. The relief is flat with a slope between 0 and 2% [22]. According to Holdridge [23], the area belongs to tropical dry forest with dominant deciduous vegetation. The soil parent material is from alluvial origin, consisting of a sandy-clay-loam texture, with kaolinite as dominant clay mineral [22]. In this region, large areas of natural forest were converted into pasture by slash-and-burn. Estrella grass (*Cynodon nlemfuensis* L.) grows for cattle use.

Specifically, the study was carried out in an area of tropical dry forest with dominant deciduous vegetation and in two adjacent pastures of 5 and 18 years old (YP and OP, resp.). The pastures were never fertilized, but annually they were cut down with machinery to control weeds and to promote grass growth. The original forest was manually cut down and burnt out, and Estrella grass was planted. Young pasture (YP) was established 5 years before starting this study. At sampling time, species of the original forest that could not be cut by hand, as well as vegetation of secondary growth, like palms and some species of legumes were observed in this pasture. The bovine cattle were introduced for grazing during dry season and at early and at end of rainy seasons. At each season the cattle remained in this pasture until they consumed the entire grass.

The old pasture (OP) was established after remotion of the original forest with machinery. Bovine cattle remained in this pasture during 3–7 days, while consuming the entire grass. The rotation time of cattle in this pasture was every 1-2 months. Weed control was similar to YP.

According to González-Pedraza and Dezzeo [24], in the study site the soils present fine texture with particular predominance of silt. Forest and OP soils show similar clay and sand contents, while YP soils have significantly higher clay content (up to 18%) than forest and OP soils. In general, the soil properties in the studied sites tended to be relatively similar (Table 1).

### 2.2. Soil Sample Collection

Soils were sampled in a natural forest and in two adjacent pastures of 5 and 18 years old. At each site, soil samples were taken from a 600 m^2^ plot (20 × 30 m). The distance between the three sites was approximately 1–3 km. At each plot, three transects were traced, and on each transect four soil samples were taken.

For determining total nitrogen in microbial biomass (Nmic), soil available nitrogen (ammonium and nitrate), and soil nitrogen mineralization, 12 soil samples were collected at 0–5 cm depth with a 5 cm diameter soil core on three periods along the year: at early rainy season (May), at end rainy season (November), and during dry season (March). To determine total nitrogen (TN), 12 samples were additionally collected at each site with the soil core at 0–5, 5–10, 10–20, 20–30, and 30–40 cm depth. These last soil samples were collected only at the end of rainy season.

### 2.3. Laboratory Analyses

The total nitrogen (TN) was determined by digestion of the samples with concentrated sulfuric acid and oxidation with hydrogen peroxide (H_2_O_2_) and colorimetrically determined by Keeney and Nelson [26] method. The ammonium and nitrate contents were extracted with a solution of potassium chloride (KCl) 2N and colorimetrically measured at a wavelength of 655 and 410 nm, respectively, following the method proposed by Keeney and Nelson [26].

Total nitrogen in microbial biomass (Nmic) was extracted by the chloroform fumigation extraction method in field moist samples [27] using 0.5N K_2_SO_4_. In these extracts, all N was converted to nitrate using the alkaline persulfate oxidation method [28] and colorimetrically determined by using the Keeney and Nelson [26] method.

Nmic was determined using the following equation:

(1) $$\begin{array}{c} \text{Nmic}=\left(\text{Nmic}\;\text{fumigated}-\text{Nmic}\;\text{unfumigated}\right)*1.85, \end{array}$$

where Nmic fumigated is total nitrogen in microbial biomass in CHCl_3_-fumigated samples and Nmic unfumigated is Nmic in nonfumigated controls.

A correction factor of 1.85 was used to account for incomplete release of microbial biomass N during the 24 h fumigation period [27].

The soil nitrogen mineralization was determined by Stanford and Smith [29] method. Every two weeks (during 15 weeks) at an incubation temperature of 35°C and under laboratory conditions, the nitrogen mineralized in soils samples was extracted with a calcium chloride (CaCl_2_) 0.01 M solution. To ensure the necessary nutrients for maintaining the activity of microbial populations during incubation time, an N free nutrient solution was used.

The ammonium and nitrate mineralized every two weeks were determined by Keeney and Nelson [26] method and the absorbance was colorimetrically measured at a wavelength of 655 and 410 nm, respectively.

From the ammonium and nitrate mineralized during 15 weeks of incubation, the N mineralized (N_*m*_), the potentially mineralizable N (N_0_), and the constant mineralization rate (*k*) were calculated according to the Stanford and Smith [29] methodology. N_*m*_ resulted of summing the ammonium and nitrate determined in each incubation interval. The cumulative N mineralized was obtained summing all N mineralized during 15 weeks of incubation. The net N mineralized every two weeks was linearly related to the square root of time through 15 weeks of incubation and N_0_ and *k* were obtained according to Stanford and Smith [29] equation:

(2) $$\begin{array}{c} {\text{N}}_{m}={\text{N}}_{0}\left(1-e^{-kt}\right), \end{array}$$

where N_*m*_ = N mineralized in time* t* (g m^−2^), N_0_ = potentially mineralizable N (g m^−2^), *k* = mineralization rate constant of first-order kinetics (weeks^−1^), and* t* = time of incubation (weeks).

Total N, Nmic, available, and mineralized N (ammonium and nitrate) values were corrected to dry soil and the results were expressed in g N-NH_4_^+^ and g N-NO_3_^−^ by m^−2^, based on the soil bulk density (kg m^−3^) and depth (m). Nitrogen losses were calculated by comparing the values found in pastures with the original forest values.

### 2.4. Statistical Analysis

Statistical analysis of data was carried out by an analysis of variance (ANOVA). The soil clay content showed significant differences between forest and pastures (Table 1), so it was used as a covariable to adjust data. Means were separated with Tukey's test when statistical differences (*P* < 0.05) were observed. When necessary, the data was transformed in order to homogenize variances, and when that did not meet this assumption (*P* > 0.05) according to Levenne's test, a nonparametric Mann-Whitney test was applied. To relate variables at sites of interest, a simple linear regression analysis was used. All statistics were computed using STATISTICA for Windows 6.0 [30].

## 3. Results

### 3.1. Total Nitrogen (TN)

**Figure 1:**
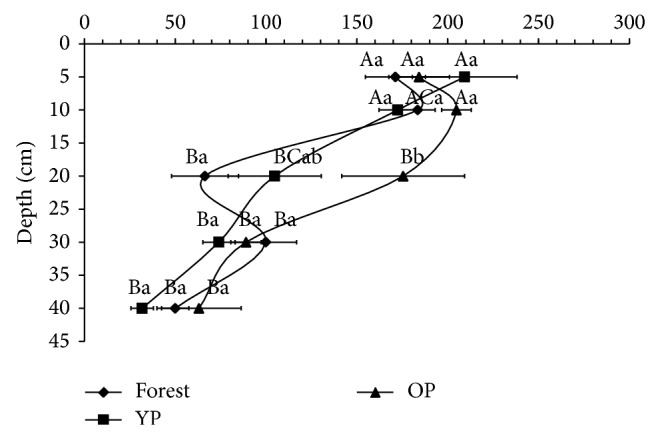
Total nitrogen (g m^−2^) in the forest and pastures soils. All points are mean values with standard error bars across forest and pastures. Different lowercase letters indicate significant differences between sites (*P* < 0.05). Different capital letters indicate significant differences between depths (*P* < 0.05). YP: 5-year-old pasture; OP: 18-year-old pasture.

No significant differences (*P* > 0.05) were detected (Figure 1) in the TN in forest, YP, and OP, with the exception of the 10–20 cm soil depths, where TN was 164.5% significantly (*P* < 0.05) higher in OP than in forest. Figure 1 also shows an uneven distribution of TN, with decreases and increases through the soil profile. It was observed that TN tends to decrease with soil depth in the three sites, except for OP at 5–10 cm soil depth and forest at 5–10 cm and at 20–30 cm soil depth.

### 3.2. Available Soil Nitrogen (Ammonium and Nitrate)

The seasonal changes of the ammonium content are shown in Table 2. At end of rainy season, the ammonium content was significantly higher (*P* < 0.05) in OP than in YP and forest. During dry season, the ammonium content was significantly higher in forest than in YP and OP. At early rainy season no statistical differences were observed in the ammonium content between sites.

Between seasons, it was noted that, with few exceptions, the ammonium content was higher at the end of the rainy season than in the dry season and at early rainy season (Table 2).

According to the results shown in Table 3, the nitrate content at the end of rainy season was lower in YP than in forest and OP. During dry season, the nitrate content was significantly higher than the value corresponding to YP, but similar to the forest value. At early rainy season, the nitrate content was significantly higher in OP than in forest and in YP, whereas between forest and YP no differences were observed.

The nitrate content in forest was higher at the end of rainy season and in dry season than at early rainy season. The nitrate content in YP was higher during dry season than in the two other seasons. In OP, no differences were observed between seasons (Table 3).

The land use change significantly affected the ammonium and nitrate content in the soils. Compared to forest soils, YP showed ammonium losses from 4.2 to 62.9% and nitrate losses from 20.0 to 77.8%, depending on the season of the year. In OP, the ammonium content increased from 50.0 to 69.0% at end of rainy season and decreased during dry season between 25.0 and 55.5%, whereas the nitrate content increased significantly at early rainy season.

### 3.3. Soil Microbial Nitrogen (Nmic)

At end of the rainy season the Nmic was significantly (*P* < 0.05) lower in YP than in forest and OP. During dry season no differences were observed between sites, while at early rainy season the Nmic was higher in OP than in forest and YP (Table 4).

Between seasons, Nmic in the forest was the highest at the end of rainy season, while between the dry season and at early rainy season no differences were observed. YP showed Nmic lower at the end of rainy season, while between dry season and early rainy season no differences were detected. Meanwhile, the Nmic in OP was significantly different (*P* < 0.05) between seasons. The highest Nmic value in OP was observed at early rainy season and the lowest value during the dry season (Table 4).

The Nmic accounted between 0.07 and 1.57% of the NT in the soils of forest, YP, and OP. At the end of the rainy season Nmic/TN ratio was significantly lower (*P* < 0.05) in YP than in forest, while the values of OP showed no differences with those of forest and YP. During dry season, no differences were observed between sites. At early rainy season the Nmic/TN ratio was significantly lower in YP than in OP, while the forest showed no differences in relation to YP and OP (Table 5).

The Nmic/TN ratio in forest soils was lower during dry season compared to the end of rainy season, while in the other sample periods no differences were observed. YP showed lower Nmic/TN ratio at the end of rainy season, and no differences were observed in the wet season. In OP the Nmic/TN ratio was lower during the dry season compared to early rainy season (Table 5).

The relationship between Nmic values with the Cmic data reported by González-Pedraza and Dezzeo [25] are shown in Table 6. According to the results, the Cmic/Nmic ratio was significantly higher in YP than in forest and OP at the end of rainy season (*P* < 0.05), while between forest and OP there were no differences. During dry season, no differences were evident between sites. However, at early rainy season the Cmic/Nmic ratio was higher in forest than in pastures.

Between seasons, a significant decrease (*P* < 0.05) in the Cmic/Nmic ratio in YP soils was observed from the end of rainy season to dry season and to the early rainy season. Forest showed no significant differences between seasons, while the Cmic/Nmic ratio in OP decreased from the dry period to early rainy season (Table 6).

### 3.4. Mineralized Ammonium and Nitrate throughout 15 Weeks of Incubation

**Figure 2:**
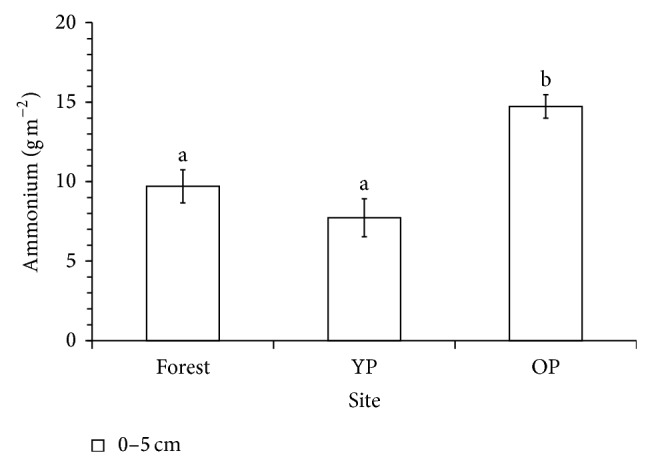
Mineralized ammonium accumulated during 15 weeks of the incubation experiment in laboratory conditions in forest and pastures soils. Mean values with standard error bars across forest and pastures. Different lowercase letters indicate significant differences between sites (*P* < 0.05). YP: 5-year-old pasture; OP: 18-year-old pasture.

**Figure 3:**
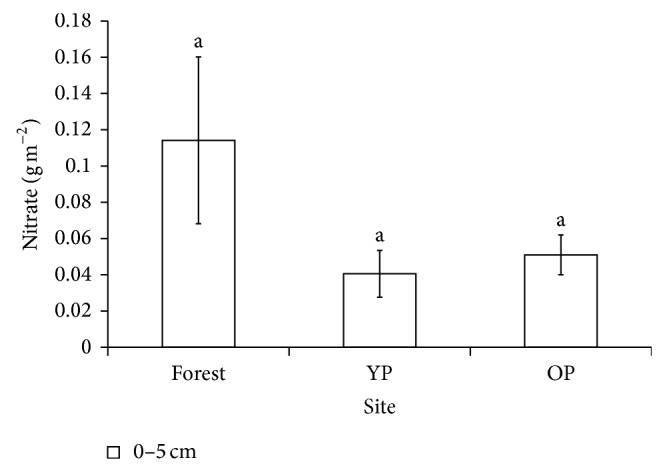
Mineralized nitrate accumulated during 15 weeks of the incubation experiment in laboratory conditions in forest and pastures' soils. Mean values with standard error bars across forest and pastures. Different lowercase letters indicate significant differences between sites (*P* < 0.05). YP: 5-year-old pasture; OP: 18-year-old pasture.

The ammonium content was statistically (*P* < 0.05) higher in OP than in forest and YP. Between forest and YP no statistical differences were found (Figure 2). The nitrate content mineralized during 15 weeks of incubation did not differ between sites (Figure 3).

### 3.5. The Net N Mineralization

The net N mineralization rate decreased during the incubation period. During the first two weeks there was a rapid N release, which was significantly higher (*P* < 0.05) in OP than in forest and YP, while between forest and YP no differences were observed. This behavior, with few exceptions, did not change throughout the incubation period. The initial flow of mineralized N during the first two weeks of incubation was 68.8% for forest, 51.9% for YP, and 56.3% for OP (Table 7).

### 3.6. Parameters of the Nitrogen Mineralization Kinetics

The N_*m*_, N_0_, and *k* mean values obtained from application of the first-order equation proposed by Stanford and Smith [29] to describe the N mineralization kinetics are given in Table 8. N_*m*_ was significantly higher (*P* < 0.05) in OP than in forest and YP, while between forest and YP no differences were observed. The *k* values varied significantly between the study sites, and the mineralized N per week ranged from 0.1 to 0.3%. In forest soils the model predicts that at 35°C and 9.1 ± 0.0 weeks (1/0.11), the obtained N_0_ (13.0 ± 4.9 g N m^−2^) was mineralized at a rate of 11% per week. In YP and OP the obtained N_0_ was mineralized at a rate of 32 and 19%, respectively (Table 8).

According to the results shown in Table 8, the estimated time by the mineralization of N_0_ was 3.12, 5.26, and 9.09 weeks for YP, OP, and forest, respectively.

In all studied sites, N_0_ was higher than N_*m*_. In that sense, it can be said that the estimated time in weeks (2.3 to 9.1 weeks) for mineralization of N_0_ in the three study sites was lower than the 15 weeks used in this experiment to obtain the N_*m*_ (Table 8). In general, the accumulated N_*m*_ and N_0_ were significantly higher (*P* < 0.05) in OP than in forest and YP. Between forest and YP no differences were found in these parameters; however, *k* was higher in YP than in forest and OP (Table 8).

### 3.7. Ammonium and Nitrate Percentage in Relation to *N*_*m*_

The ammonium content accounted around 98-99% of N_*m*_, while the nitrate content was only about 0.4 to 3.4% (Table 9). The soils of OP showed the highest N_*m*_/TN percentages (Table 10).

## 4. Discussion

### 4.1. Effect of Land Use Change on Total Nitrogen (TN)

The TN content in the soils of forest and pastures was relatively higher than that reported for other deciduous tropical forest soils [18, 31–34]. According to these results, it is clear that transformation of semideciduous dry tropical forest into pasture caused an increase in the TN, and this is not consistent with some reported data [5, 31, 35]. However, a similar trend was observed by Hassink [36], who found a higher N organic content in pastures over 10 years old than in young pastures (1–3 years old) and forest soils.

In many cases the management conditions can exert an important role in the soil nitrogen dynamic, especially if the pastures are fertilized. The pastures in the study site were never fertilized and they are not subjected to overgrazing. However, it is important to emphasize that star grass (*Cynodon nlemfuensis* L.) is a species with stoloniferous growth and abundant roots production. According to observations made in OP soils during the field sampling, star grass formed a layer on the ground composed by leaves and remnant stems, which could enhance the soil organic matter and, consequently, the total nitrogen of soil. Another factor that could explain the enhanced soil nitrogen, especially in YP, is related to an additional input of organic matter from the remaining plants of the original forest and from the secondary forest vegetation that continued regrowth.

The soil organic matter is the main N source in the soils [37], and it exerts an important effect on the dynamics of this element into the soil. According to González-Pedraza and Dezzeo [25], the soil organic carbon (SOC) for the studied sites was higher in pastures than in forest (Table 1). Additionally, the positive correlations found between SOC data and TN in forest and OP (*r* = 0.82 and 0.65, resp.) evidenced that the TN is closely related to the SOC in these sites. Therefore, the factors affecting the SOC help to explain the dynamics of TN in these soils.

The soil texture is another factor that could influence the TN behavior. According to González-Pedraza and Dezzeo [24], the YP soil showed higher clay percentage (%C) compared to forest. In that sense, a correlation was made between the TN and %C data, and a positive correlation in YP (*r* = 0.73, *P* < 0.05) was found. This indicates that the clay in the soil is protecting the soil nitrogen through organic matter. Similar results were reported by Hassink [36], who found a positive relationship between the organic N and the clay content.

### 4.2. Effect of Seasonality and Land Use Change on Available Nitrogen (Ammonium and Nitrate)

The land use change significantly affected the ammonium and nitrate content in soils. As mentioned in the results, YP showed ammonium and nitrate losses compared to the forest, while OP showed nitrate increases and ammonium increases or decreases, depending on the sample period. Similar results were reported by Johnson and Wedin [5], Neill et al. [10], and Ellingson et al. [20], who found that in pastures, especially young pastures, the ammonification and nitrification are lower than in original forest. During early stages of the pastures establishing, the large losses of nitrate have been associated with volatilization, erosion, and leaching due to a decrease in the absorption rate by vegetation and immobilization of soil microorganisms [1, 6, 9].

N availability also has been positively correlated with the C and N levels in savannas, pastures, and agricultural crops' soils [38]. For example, Johnson and Wedin [5] pointed out that in contrast to the low efficiency in the use of nutrients that characterize woody species in tropical forests, perennial grasses, particularly those adapted to fire, generally have a high C/N ratio in senescent aerial and underground tissues, allowing them to immobilize N during decomposition. García-Oliva et al. [31] and Hassink [36] also found higher C/N ratio in pastures than in forest soils.

The pastures age is another important factor affecting the nitrogen availability in the soil. In this study, the general trend towards an increase in the ammonium and nitrate content in relation to the pasture age is comparable with the data reported by Hassink [36], who found an increase in the total available N and a decrease in the C/N ratio in pastures soils with over 10 years old than in those between 1 and 3 years old.

The N transformation in the soils is mainly mediated by microorganisms [39, 40], and the microbial activity depends mainly on the substrate quantity, quality, and availability. It also depends on temperature and soil humidity [6, 41]. In this study, the ammonium and nitrate content increased with the increasing substrate availability and microbial activity, as indicated by Booth et al. [41]. Likewise, the nitrate content increase in pastures soils has been associated with a higher microbial transformation of ammonium and a low rate of absorption by grass plants [20]. In addition, it is possible that in OP the best quality of the substrate and major humidity percentage stimulated the microbial activity compared to forest.

The soil moisture content has a positive effect on the ammonium and nitrate content in the soils. [8, 42, 43]. During rainy season, the environmental conditions promote the activation of soil microbial processes [43, 44], which explains the higher ammonium content found in this study at the end of this season. It is very important to point out that in the western Llanos, the wet season ranges from 6 to 8 months along the year, and the samples of this study were taken at the end of rainy season, which favored the soil N availability, especially ammonium.

However, it is probable that at early rainy season both the ammonium and nitrate could be lost due to lixiviation and runoff. It is also possible that during the growing season, usually associated with the rainy months, a greater plant uptake occurs. This, perhaps, may explain the significant low contents found at early rainy season in relation to those at end of rainy season and during the dry period. In addition, it is probable that with the beginning of the rain the competition between soil microorganisms and plants to take the available nutrients increases, causing a decrease in the soil nitrogen availability.

On the other hand, the nitrate content increase evaluated during dry season in the three sites could be attributed to a decrease in the plant nutrient demand and also to microbial death [45].

### 4.3. Effect of Seasonality and Land Use Change on Soil Microbial Nitrogen (Nmic)

Land use change and the seasonality in the precipitation regimen affected the Nmic behavior. Nmic values found in this study are lower than those reported for tropical dry forests, cultivated pastures, and savannas [8, 31, 44, 45]. However, these values are similar to those obtained by Jensen et al. [34] for savanna soils. Meanwhile, the Nmic/TN ratio values are lower than those reported by García-Oliva et al. [31] and by Jensen et al. [34] for areas with similar climatic conditions and vegetation.

For the study area, González-Pedraza and Dezzeo [25] pointed out that the microbial populations respond differently to changes in the soil moisture content throughout the year. These authors also showed that soil microbial activity was affected by vegetation type, seasonality of rainfall, and pasture age. This could indicate possible physiological differences between microbial communities in the three study sites, which can be associated with the fact that each site responded differently to changes in soil moisture content. González-Pedraza and Dezzeo [25] concluded that in YP soils, microorganisms were apparently less efficient to decompose the organic matter, while in OP the best quality of the substrate stimulated the microbial activity. Besides, the old age of OP allowed greater stability of soil microbial activity.

It is possible that the higher Nmic shown in OP at early rainy season could be related to the fact that the organic matter in this period was more palatable and had an easier decomposition for microbial populations, which allows it to be more active [46–51]. The Nmic increase in YP and decrease in forest and OP during the dry season reflect that the microbial populations respond differently to moisture content variations.

The Nmic decrease during dry season and increase with early rains can be associated with the microbial activation after the low activity during dry season due to the reduced soil moisture availability [52]. Additionally, at early rainy season many nutrients from the microbial biomass and soil organic matter are solubilized, becoming more accessible to the microorganisms [52].

Microbial biomass activity depends on factors such as humidity, temperature, soil texture, and the quantity and quality of plant material used as a substrate [47, 49, 51–56]. In this study, the Nmic in forest soil was significantly and positively correlated with the ammonium content (*r* = 0.95; *P* < 0.05) during dry season. On the other hand, at early rainy season Nmic was positively correlated with both the clay percentage (*r* = 0.84; *P* < 0.05) and the ammonium content (*r* = 0.90; *P* < 0.05).

The Nmic in pasture soils was related to the Cmic data reported by González-Pedraza and Dezzeo [25] and showed a high Cmic/Nmic ratio, especially at end of rainy season and during dry season. This result clearly shows differences in the composition of microbial communities on these soils. For example, the high Cmic/Nmic ratio found in YP soils at the end of rainy season reflects a strong limitation of available N during this season. This is also supported by the low content of TN, ammonium, and nitrate found in YP (Table 5).

In addition, a negative correlation (*r* = −0.68; *P* < 0.05) was found between the ammonium content and Nmic in YP soils. This could be due to a low substrate quality which could inhibit the metabolic process of soil microorganisms. Otherwise, in YP soils the microorganisms are, apparently, less efficient to decompose the organic matter and mineralize soil nitrogen [25]. It is also probable that the TN pool in YP can be immobilized in plant tissues due to stress conditions, such as defoliation by cattle, and therefore it could be inaccessible to microorganisms [53]. As a consequence, the YP showed low contents of available N and Nmic.

At the end of the rains the Nmic was lower in the pasture than in forest soil, contrary to what happened in dry season. This suggests that at the end of rainfall the available N fractions are immobilized by microorganisms, absorbed by plants, or lost by leaching and/or erosion.

### 4.4. Effect of Seasonality and Land Use Change on Soil Nitrogen Mineralization

According to the results shown in Table 9, in the three studied sites ammonium was the predominant form of N mineralized during 15 weeks of incubation, while nitrate was very low both in the forest and pastures. This indicates that ammonification was the main privileged process, not nitrification. It is probable that the nitrification can be inhibited under slight acidic soils with soil pH < 6.0 [24], as those of the study area.

The N_*m*_/TN values are higher than those reported by Arias de Estrada [56] for some dry tropical forest and pasture soils in western Llanos (Portuguesa and Barinas states), but relatively similar to those found by Sánchez et al. [57] for deciduous dry tropical forest and savannas of central Llanos (Guárico state). The highest N_*m*_/TN found in OP would indicate that these soils are more able to mineralize nitrogen than forest and YP.

The land use change from forest to pasture affected the soil nitrogen mineralization. The mineralization pattern appears to be similar in the three sites, but the mineralization magnitude was different between forest and pastures. More than 50% of the available N into the soil was mineralized at the first two weeks of incubation. This accelerated flow has been associated with drying and physical disturbance of the soil samples, which cause death of a portion of the microbial population and their rapidly mineralization when soil is rewetting [52, 58, 59]. This could also be associated with the presence of N forms susceptible of being decomposed. Besides, the physical disturbance of the soil organic matter due to handling can contribute to accelerating the nitrogen mineralization of compounds highly sensitive to this process [59–61].

Because of depletion of the more labile soil organic matter fraction, the net N mineralization decreased in relation to the incubation time. This has been associated with the decrease of the potentially mineralizable N and the microbial population size during incubation time under laboratory conditions [61].

The N_*m*_ found in OP soils was significantly higher than that in forest and YP. Although no statistical differences were found in the TN values between sites, OP had 22.3% more TN than the forest. In addition, OP also presented the highest N_*m*_/TN ratio, which would indicate that N in this pasture is more available for being mineralized than in forest and in YP. Moreover, it is probable that this old pasture could have a gradual and prolonging nutrient cycling in the ecosystem, which could allow more efficiency in the use of this substrate by the soil microorganisms.

In a previous section it was discussed that in pastures with a longer established time, an increase in the total N content and mineralization rate [36] can occur. It was also mentioned that the ammonium and nitrate mineralization was higher with increases in the substrate availability and in the microbial activity [41].

The N_0_ in OP was 42.3% higher than in forest. This clearly indicates that in OP there was a larger pool of potentially available N. This could be associated with the pasture established time. Higher values of N_*m*_ and N_0_ found in OP could also be indicating a better quality substrate for mineralization in pastures soils.

The relatively N_0_ low values found in YP are, probably, due to a lower efficiency of microorganisms to compete with plants for nitrogen demand. Similarly, the short establishment time and the continuous pasture grazing have, probably, led to a depletion of available soil N because the microorganisms are highly selective and they use first labile forms of organic matter.

Because of a lower substrate quality, the high *k* values found in YP could be related to a reduced ability to preserve the available nitrogen in these soils resulting in a grass survival strategy under those conditions. In this sense, although YP had a lower N_0_, its mineralization rate was higher.

## 5. Conclusions

Land use change from forest into pasture led to an increase in TN in almost all soil depths. Among the pastures, OP had a greater TN content than YP. The TN increase in YP topsoil was influenced by clay percentage. In OP, the established time has allowed a greater input and easier organic matter degradation, reflected in a higher content of N into the soil. During the three seasons, soil ammonium mineralization was the most important process in these soils resulting in the highest proportion of this nitrogen form in relation to TN.

The pasture establishment led to a decrease of ammonium and nitrate content and also to a decrease of soil microbial activity. The youngest pasture presented lower values of ammonium, nitrate, Nmic, N_0_, and N_*m*_ in comparison with forest and old pasture. This pasture also had a higher mineralization rate (*k*). This leads to the conclusion that after the forest cutting and burning, the initial establishment of pastures negatively impacted on the soil N dynamics. This is reflected in the decrease of quality substrate and microorganisms efficiency to use this substrate. Therefore, the capacity of preserving the available N is reduced, and this can lead to N exhaustion. The higher values of N_*m*_ and N_0_ found in OP are indicating a better quality of the substrate in this pasture.

Seasonality in precipitation had a marked effect on the soil N dynamics in the studied site. Prolonged rainy season privileged microbial processes that allowed a greater ammonium and nitrate mineralization and a higher Nmic. At early rainy season, ammonium and nitrate losses were associated with leaching and runoff caused by the first rainfall and by a nutrient demand increase for vegetation to grow. The nitrate increase during dry season was associated with the lower nutrient demand of plants and microorganisms and with microbial population decrease in the three evaluated sites.

## Figures and Tables

**Table 1:** Soil properties in the study sites (according to González-Pedraza and Dezzeo [24, 25]).

Component	Depth (cm)	Forest	YP	OP
Bulk density (g cm^−3^)	0–5	1.2 ± 0.0^a^	1.1 ± 0.1^a^	1.1 ± 0.0^a^
%Humidity	0–5	31.4 ± 1.0^a^	37.9 ± 1.7^b^	33.9 ± 0.9^ab^
%Sand	0–5	19.8 ± 1.7^a^	12.5 ± 2.3^b^	22.3 ± 1.6ª
%Silt	0–5	49.7 ± 1.8^a^	44.8 ± 1.8^a^	47.1 ± 1.3^a^
%Clay	0–5	30.5 ± 2.8^a^	42.7 ± 3.1^b^	30.6 ± 1.9^a^
pH H_2_O	0–5	5.4 ± 0.1^a^	5.0 ± 0.14^ab^	4.8 ± 0.1^b^
SOC (g C m^−2^)	0–5	1389.9 ± 251.9^a^	1472.7 ± 400.2^a^	1516.7 ± 249.9^a^

Mean values ± standard deviation. Different lowercase letters indicate significant differences between sites (*P* < 0.05). YP: 5-year-old pasture; OP: 18-year-old pasture; SOC: soil organic carbon.

**Table 2:** Seasonal changes in the available soil ammonium in the forest and pastures.

Season	Depth (cm)	Available ammonium (N-NH_4_^+^), g m^−2^		
		Forest	YP	OP
End rainy season	0–5	3.3 ± 1.7^Aa^	2.8 ± 1.1^Aa^	5.6 ± 0.9^Ab^
Dry season	0–5	2.7 ± 1.0^Aa^	1.0 ± 0.3^Bb^	1.2 ± 0.3^Bb^
Early rainy season	0–5	0.7 ± 0.2^Ba^	0.6 ± 0.1^Ba^	0.7 ± 0.2^Ba^

Mean values ± standard deviation. Different lower case letters indicate significant differences between sites (*P* < 0.05). Different capital letters indicate significant differences between seasons (*P* < 0.05). YP: 5-year-old pasture; OP: 18-year-old pasture.

**Table 3:** Seasonal changes in the available soil nitrate in the forest and pastures.

Season	Depth (cm)	Available nitrate (N-NO_3_^−^), g m^−2^		
		Forest	YP	OP
End rainy season	0–5	0.9 ± 0.7^Aba^	0.2 ± 0.2^Ab^	1.4 ± 0.5^Aa^
Dry season	0–5	1.4 ± 1.0^Aab^	0.5 ± 0.3^Ba^	1.7 ± 1.2^Ab^
Early rainy season	0–5	0.5 ± 0.1^Ba^	0.4 ± 0.2^ABa^	1.0 ± 0.3^Ab^

Mean values ± standard deviation. Different lowercase letters indicate significant differences between sites (*P* < 0.05). Different capital letters indicate significant differences between seasons (*P* < 0.05). YP: 5-year-old pasture; OP: 18-year-old pasture.

**Table 4:** Soil microbial nitrogen (Nmic) in the forest and pastures.

Season	Depth (cm)	Nmic (g m^−2^)		
		Forest	YP	OP
End rainy season	0–5	2.4 ± 1.2^Aa^	0.2 ± 0.1^Ab^	1.4 ± 0.3^Aa^
Dry season	0–5	0.7 ± 0.7^Ba^	1.1 ± 0.6^Ba^	1.0 ± 0.7^Ba^
Early rainy season	0–5	1.3 ± 0.4^Ba^	1.2 ± 0.5^Ba^	2.0 ± 0.5^Cb^

Mean values ± standard deviation. Different lowercase letters indicate significant differences between sites (*P* < 0.05). Different capital letters indicate significant differences between seasons (*P* < 0.05). YP: 5-year-old pasture; OP: 18-year-old pasture.

**Table 5:** Microbial nitrogen percentage in the total soil nitrogen in forest and pastures.

Season	Depth (cm)	Nmic/TN (%)		
		Forest	YP	OP
End rainy season	0–5	1.3 ± 0.9^Aa^	0.2 ± 0.1^Ab^	0.7 ± 0.4^Aab^
Dry season	0–5	0.5 ± 0.5^Ba^	0.7 ± 0.4^Ba^	0.5 ± 0.4^Aa^
Early rainy season	0–5	0.8 ± 0.4^ABab^	0.6 ± 0.3^Ba^	1.1 ± 0.3^Bb^

Mean values ± standard deviation. Different lowercase letters indicate significant differences between sites (*P* < 0.05). Different capital letters indicate significant differences between seasons (*P* < 0.05). Nmic: microbial nitrogen; TN: total nitrogen; YP: 5-year-old pasture; OP: 18-year-old pasture.

**Table 6:** Relationship between soil microbial carbon and soil microbial nitrogen in the forest and pastures.

Season	Depth (cm)	Cmic/Nmic		
		Forest	YP	OP
End rainy season	0–5	16.1 ± 7.2^Aa^	169.4 ± 68.1^Ab^	27.6 ± 9.5^Aba^
Dry season	0–5	29.9 ± 29.3^Aa^	34.1 ± 25.6^Ba^	36.4 ± 16.0^Aa^
Early rainy season	0–5	26.5 ± 8.3^Aa^	16.2 ± 9.7^Bb^	16.1 ± 7.4^Bb^

Mean values ± standard deviation. Different lowercase letters indicate significant differences between sites (*P* < 0.05). Different capital letters indicate significant differences between seasons (*P* < 0.05). YP: 5-year-old pasture; OP: 18-year-old pasture. Cmic: microbial carbon; Nmic: microbial nitrogen.

**Table 7:** Net nitrogen mineralized by week throughout 15 weeks of incubation in the soil forest and pastures.

Week	Depth (cm)	Net nitrogen mineralized, g m^−2^		
		Forest	YP	OP
2	0–5	2.4 ± 0.6^a^	2.1 ± 1.0^a^	4.0 ± 0.4^b^
4		4.4 ± 1.7^a^	1.9 ± 1.8^b^	4.3 ± 2.6^a^
6		0.8 ± 0.5^a^	1.1 ± 0.8^a^	2.4 ± 0.5^b^
8		1.1 ± 0.4^a^	0.9 ± 0.7^a^	1.9 ± 0.6^b^
10		0.6 ± 0.3^a^	0.9 ± 0.4^ab^	1.1 ± 0.4^b^
12		0.4 ± 0.2^a^	0.6 ± 0.3^a^	0.8 ± 0.2^b^
15		0.2 ± 0.3ª	0.2 ± 0.2^a^	0.3 ± 0.3^a^

Mean values ± standard deviation. Different lowercase letters indicate significant differences between sites at the same week (*P* < 0.05). YP: 5-year-old pasture; OP: 18-year-old pasture.

**Table 8:** Parameters derived from the N mineralization kinetics by applying the first-order equation N_*m*_ = N_0_[(1 − exp⁡^−*kt*^)].

Parameter from the N mineralization kinetics	Depth (cm)	Forest	YP	OP
N_*m*_ (g m^−2^)	0–5	9.8 ± 3.5^a^	7.8 ± 4.2^a^	14.8 ± 2.6^b^
*k* (1/week)	0–5	0.1 ± 0.0^a^	0.3 ± 0.2^b^	0.2 ± 0.1^a^
N_0_ (g m^−2^)	0–5	13.0 ± 4.9^a^	10.1 ± 5.3^a^	18.5 ± 4.7^b^
*R* ^2^	0–5	0.97	0.93	0.96

Mean values ± standard deviation. Different lowercase letters indicate significant differences between sites (*P* < 0.05). YP: 5-year-old pasture; OP: 18-year-old pasture. N_*m*_ = mineralized nitrogen accumulated during 15 weeks of incubation, *k* = constant rate of nitrogen mineralization, N_0_ = potentially mineralizable nitrogen, and *R* ^2^ = coefficient of the determination.

**Table 9:** Relationship between soil ammonium and nitrate in relation to the total mineralized N accumulated throughout 15 weeks of incubation in forest and pastures.

Parameter	Depth (cm)	Site		
		Forest	YP	OP
%Ammonium/N_*m*_	0–5	98.2 ± 3.7^a^	99.4 ± 0.6^a^	99.6 ± 0.3^a^
%Nitrate/N_*m*_	0–5	1.8 ± 3.7^a^	0.6 ± 0.6^a^	0.4 ± 0.3^a^

Mean values ± standard deviation. Different lowercase letters indicate significant differences between sites (*P* < 0.05). YP: 5-year-old pasture; OP: 18-year-old pasture. N_*m*_ = mineralized nitrogen accumulated throughout 15 weeks of incubation.

**Table 10:** Percentage of the mineralized N accumulated in relation to total nitrogen soil in forest and pastures.

	Depth (cm)	Site		
		Forest	YP	OP
N_*m*_/NT (%)	0–5	6.1 ± 2.3^ab^	4.6 ± 3.9^a^	8.5 ± 2.6^b^

Mean values ± standard deviation. Different lowercase letters indicate significant differences between sites (*P* < 0.05). YP: 5-year-old pasture; OP: 18-year-old pasture. N_*m*_ = N mineralized accumulated; TN = total nitrogen.
